# Germanium stable isotope measurements by double-spike MC-ICPMS

**DOI:** 10.1039/d4ja00359d

**Published:** 2025-02-27

**Authors:** Elias Wölfer, Christoph Burkhardt, Thorsten Kleine

**Affiliations:** a Max Planck Institute for Solar System Research Justus-von-Liebig-Weg 3 37077 Göttingen Germany woelfer@mps.mpg.de

## Abstract

We present analytical procedures for the measurement of mass-dependent Ge isotope compositions using a ^70^Ge–^73^Ge double spike and multiple-collector inductively coupled plasma mass spectrometry (MC-ICP-MS). Two different mass spectrometers (ThermoScientific Neptune Plus and Neoma) and two different sample introduction systems (Teledyne Cetac Technologies HGX-200 hydride generator and, for the first time in Ge isotope analyses, a Cetac Technologies Aridus II desolvator) were used. A series of analytical tests demonstrate that our analytical procedure efficiently separates Ge from the sample matrix and provides accurate and precise Ge concentration and isotope data for both instruments and sample introduction methods. The external reproducibility (2 s.d.) of the entire analytical procedure is ±0.09‰ for *δ*^74/70^Ge (the permil deviation from the National Institute of Standards and Technology (NIST) Standard Reference Material (SRM) 3120a). Using the new methods, we obtained Ge isotope data for an Alfa Aesar Ge standard solution, the NIST SRM metals 129c and 261, two terrestrial basalts (BHVO-2 and BCR-2), and eight iron meteorites. We find excellent agreement between the data obtained using the hydride generator and using the Aridus II desolvator. Due to the overall easier use of the latter, the desolvator may be the sample introduction system of choice for most Ge isotope applications. Overall, the samples of this study show *δ*^74/70^Ge variations up to ∼2‰, and in agreement with literature data, indicate that mass-dependent Ge isotope variations may be used to study a wide range of geochemical and cosmochemical processes.

## Abstract 2

Comparison of *δ*^74/70^Ge data obtained for different samples using (A) either an Aridus desolvator or a hydride generator, or (B) either a Neptune Plus or a Neoma MC-ICP-MS. Both introduction systems and mass spectrometers yield consistent results.

## Graphical abstract

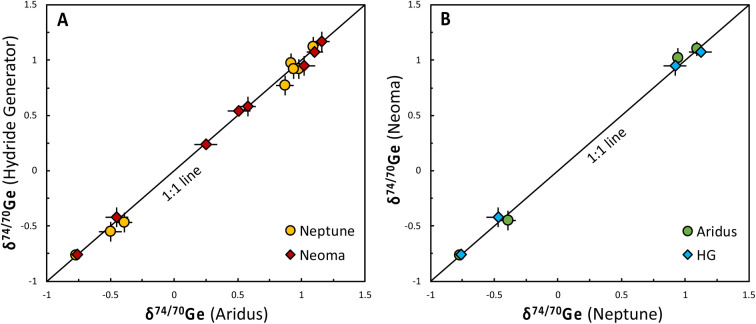

## 1 Introduction

The isotopic and elemental compositions of planetary materials are sensitive recorders of the physicochemical conditions prevailing during their formation and hence are versatile tracers of geological and astrophysical processes throughout the history of the solar system.^1–4^ For instance, determining the magnitude and direction of elemental and stable isotope fractionations among terrestrial samples and meteorites can provide information about the nature and conditions of the processes involved in the formation and internal evolution of asteroids and planets.^5–8^ Germanium (Ge) has five naturally occurring isotopes (^70^Ge, ^72^Ge, ^73^Ge, ^74^Ge, and ^76^Ge) and is a promising element to study stable isotope (and elemental) variations among natural samples.^9,10^ It is a moderately volatile element (MVE, 50% condensation temperature ∼883 K),^11,12^ belongs to the group of semimetals, and behaves moderately siderophile during metal-silicate fractionation processes such as planetary core formation.^9,10,13^ Moreover, under lower pressure and temperature conditions, Ge can become highly siderophile,^14^ whereas it behaves as a lithophile under oxidizing conditions and chalcophile in sulfur-rich environments.^13^ This variable geochemical behavior of Ge is evident from the range of oxidation states it assumes in natural samples. Germanium occurs as Ge^0^ in metals (*e.g.*, iron meteorites), Ge^2+^ in sulfides (*e.g.*, as GeS in pyrite, galena, and sphalerite), and Ge^4+^ in the major rock-forming, silicate minerals (*e.g.*, pyroxene and olivine). Overall, the range of oxidation states of Ge, together with its relatively volatile behavior and variable geochemical character, makes Ge isotope fractionations in many geologically relevant environments likely.

In terrestrial settings, mass-dependent Ge isotope variations have been investigated in a number of studies.^9,10,15–21^ These studies have revealed that the silicate rocks of the Earth's crust and mantle are depleted in Ge as a result of core formation and show rather homogeneous *δ*^74/70^Ge values (*i.e.*, the permil deviation of the ^74^Ge/^70^Ge ratio from a standard), indicating there is only limited Ge isotope fractionation during melting and fractional crystallization processes within the silicate Earth. By contrast, Ge is highly enriched and isotopically fractionated in sphalerite and other sulfide deposits, most likely as a result of kinetic isotope fractionation during sulfide formation.

In the field of cosmochemistry, Ge isotopes have not yet attracted major interest, despite their broad potential range of use. With the exception of ordinary chondrites,^22^ the unusual CB chondrites,^23^ and some iron meteorites groups,^16,17,24^ no mass-dependent Ge isotope data have been published. Nevertheless, the available data alone already demonstrate that Ge isotopes are fractionated in meteorites by more than 2‰, highlighting the potential of Ge stable isotopes for examining processes associated with the formation and subsequent evolution of meteorites and their parent bodies. The scarcity of Ge isotope data for meteorites at least in part reflects analytical difficulties associated with Ge isotope measurements by multicollector inductively coupled plasma mass spectrometry (MC-ICPMS). For instance, earlier studies on meteorites used a cyclonic spray chamber as the sample introduction system, but this method requires relatively large amounts of Ge, thereby limiting the samples that can be analyzed to some Ge-rich iron meteorites.^16,17^ More recent studies overcame this problem by using a hydride generator for the sample introduction, which improves sensitivity and thus allows for measurements of Ge-poorer samples.^21–23^ However, until now only a small number of meteorite samples have been analyzed using this technique.

To facilitate Ge isotope measurements for a comprehensive set of extraterrestrial samples irrespective of their Ge concentration and modal mineralogy (*i.e.*, silicate- and metal-dominated samples), we developed analytical procedures for precise Ge isotope measurements by ^70^Ge–^73^Ge double spike MC-ICPMS, using either a Cetac Technologies Aridus II desolvator or a Teledyne Cetac Technologies HGX-200 Hydride Generator as the sample introduction systems. Compared to prior studies, our analytical procedures include several important novel aspects. First and foremost, a desolvator as a sample introduction system has so far not been used for Ge isotope measurements, but we will show below that this may be the method of choice for most Ge isotope applications. Second, the application of a Ge double spike has yet mostly been limited to geothermal fluids and (sea)water samples.^21,25–27^ With the exception of a single study on terrestrial rocks^18^ and a single study using thermal ionization mass spectrometry,^28^ no Ge double spike has been used for precise Ge isotope measurements in planetary sciences. Third, direct comparison of Ge isotope data obtained using different introduction systems is very limited. Finally, our methods, which are tailored towards the analyses of meteorites, will allow precise Ge isotope measurements of strongly Ge-depleted iron meteorites, which have not been possible using previously used techniques.^16,17^ Together, the new analytical procedures presented here will, therefore, help to unlock the full potential of Ge isotopes as a tracer in geochemistry and cosmochemistry.

## 2 Germanium double spike – design and preparation

The double spike technique is a precise measurement technique for determining mass-dependent isotope variations in natural samples.^29–31^ The major advantage of using a double spike compared to the conventional standard-sample method is that the natural mass-dependent isotope fractionation (*i.e.* the quantity we want to determine) can be distinguished from mass-dependent isotope fractionation induced during sample preparation and measurement. This makes double spike measurements less prone to potential analytical artifacts related to different sample matrices, incomplete recovery during chemical separation, and variable measurement conditions (*e.g.*, drifts in instrumental mass fractionation).

The reduction of double spike data relies on the assumption that any isotopic difference between the sample and the standard is purely mass-dependent.^29,32^ However, especially among extraterrestrial samples, mass-independent isotope anomalies are widespread (such as *e.g.* nucleosynthetic anomalies), and the presence of such anomalies might lead to spurious results, if not corrected for.^33,34^ However, until now no such anomalies have been reported for Ge. This is consistent with our finding that all meteorite samples analyzed in this study using a Ge double spike show indistinguishable mass-dependent Ge isotope compositions compared to previous measurements on the same samples obtained without the use of a Ge double spike (see Fig. 5 and 8 and Section 4.4 for details). Therefore, mass-independent Ge isotope anomalies either do not exist or, if present, are small and as such have no measurable effect on the mass-dependent Ge isotope data reported here.

**Fig. 1:**
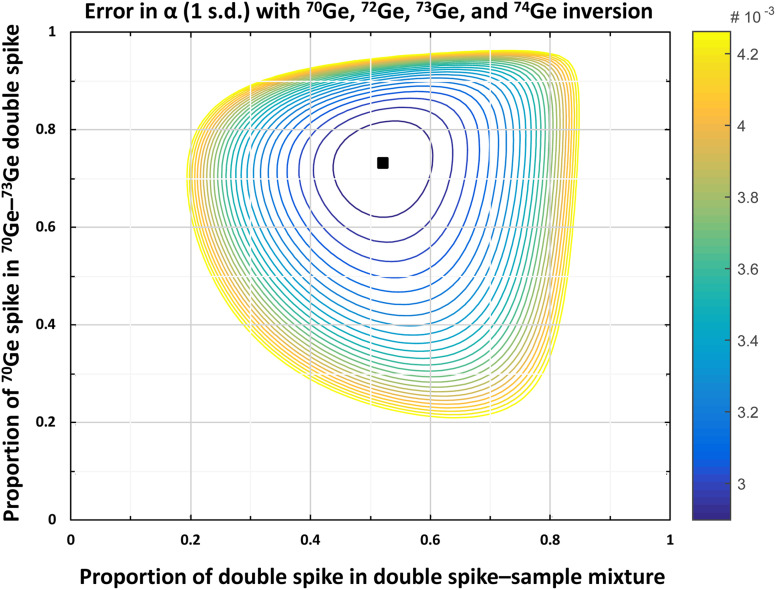
Contour plot of the error propagation on the natural fractionation coefficient *α* (1 s.d.) in the ^70^Ge, ^72^Ge, ^73^Ge, and ^74^Ge inversion depending on the proportion of double spike in the double spike–sample mixture and the proportion of ^70^Ge spike in the ^70^Ge–^73^Ge double spike as calculated using the Double Spike Toolbox.^29^ The calculated optimum is marked with a black square (*i.e.*, 73.18% ^70^Ge spike in the ^70^Ge–^73^Ge double spike; 52.15% double spike in the double spike–sample mixture). The contour lines represent a 1% increase in the error on α relative to the minimum error.

**Fig. 2:**
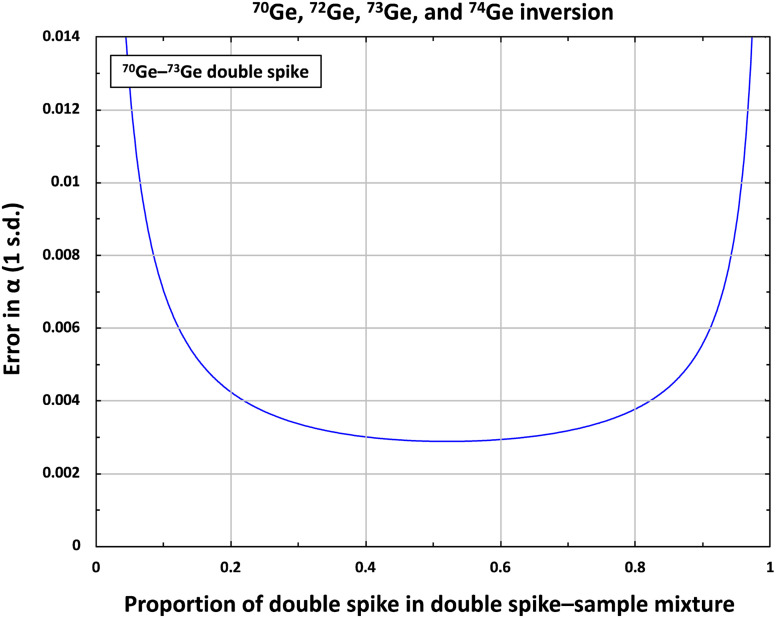
Propagation of the theoretical uncertainties (error) on the natural fractionation coefficient *α* as a function of the proportion of Ge double spike in the spike–sample mixture calculated using the Double Spike Toolbox and using ^70^Ge, ^72^Ge, ^73^Ge, and ^74^Ge for the inversion.^29^

Traditional double spike measurements use four different isotopes, two of which are the spike isotopes, which together with the other two unspiked isotopes are used in the double spike inversion to determine the natural mass fractionation factor α.^29,35^ Germanium has five naturally occurring stable isotopes (^70^Ge, ^72^Ge, ^73^Ge, ^74^Ge, and ^76^Ge), and there are several options for the setup of a Ge double spike. However, ^76^Ge should be avoided because of Ar-based polyatomic interferences (*e.g.*, ^36^Ar^40^Ar and ^36^S^40^Ar) at mass 76, which naturally occur in MC-ICPMS measurements using an Ar plasma. Similar to prior studies using a Ge double spike,^18,21,25–27^ of the remaining four Ge isotopes, we selected the two less-abundant isotopes ^70^Ge (20.57%) and ^73^Ge (7.75%) for the double spike, which was optimized using ^70^Ge, ^72^Ge, ^73^Ge, and ^74^Ge in the inversion. The predicted intrinsic error on α of the ^70^Ge–^73^Ge double spike setup is small [∼39 ppm per amu; 1 s.d.],^29^ whereas the range of permissible double spike–sample ratios is relatively large (Fig. 1 and 2). All other possible double spike designs using ^70^Ge, ^72^Ge, ^73^Ge, and ^74^Ge would result in both larger errors on *α* and a smaller range of permissible double spike–sample ratios.

The ^70^Ge and ^73^Ge single spikes (batch no. 198640 and 198 841, respectively) were obtained as ∼10 mg fine grained metal powder from the Oak Ridge National Laboratory. The powders were weighed into 15 ml Savillex Teflon perfluoroalkoxy (PFA) vials and digested *via* table-top digestion using 10 ml 8 M HNO_3_–0.1 M HF at 120 °C for 10 days. After complete digestion, the solutions were transferred into larger Savillex PFA vials and diluted with MQ. Small aliquots of the two single spikes were then used for determining their Ge concentrations and isotopic compositions by MC-ICPMS. The two single spikes were then combined into the final ^70^Ge–^73^Ge double spike, where the mixing proportions were optimized to minimize the error propagation on *α* (73.18% ^70^Ge spike and 26.82% ^73^Ge spike, Fig. 1). The double spike was dried down, re-dissolved in 0.78 M HNO_3_–0.01 M HF, and equilibrated for 48 h at 120 °C on a hotplate. Finally, small aliquots of the double spike were used to determine its isotopic composition and concentration by MC-ICPMS (see below).

## 3 Materials and methods

### 3.1 Chemicals and standard solutions

All laboratory work was conducted in class-10 000 clean room environments using class-10 laminar flow hoods at the Institut für Planetologie, University of Münster, and the Max Planck Institute for Solar System Research in Göttingen. Pre-cleaned Savillex PFA vials and bottles were used for all samples and solutions processed in this study. Merck Millipore Emsure grade acids (69% HNO_3_, 48% HF) were distilled twice using Savillex DST-1000 acid purification systems. Diluted acids were produced using Merck Millipore Milli-Q water (18.2 MΩ cm).

The National Institute of Standards and Technology (NIST) Standard Reference Material (SRM) 3120a Ge solution standard (Lot no. 080429; 10.015 ± 0.021 mg per g Ge) is the recommended standard reference solution for stable Ge isotope measurements,^17,18^ and was used as the reference standard throughout this study. We routinely also measured an Alfa Aesar Specpure 1000 μg per ml Ge plasma standard solution in 5% HNO_3_/traces of HF (Lot no. 12-12328C) along with each sample set to monitor the accuracy and reproducibility of our analytical method.

### 3.2 Geological samples

To test the accuracy and reproducibility of our method, different geochemical NIST reference materials were analyzed. These include the metal reference materials NIST 129c and NIST 361 to test the functionality of the chemical separation of Ge from metal samples, as well as the terrestrial basalts BHVO-2 and BCR-2 provided by the United States Geological Survey (USGS), to test the functionality of the chemical separation of Ge from silicate samples.

To investigate the magnitude and direction of Ge stable isotope variations in extraterrestrial samples, we analyzed three variably Ge-depleted magmatic iron meteorites from both the non-carbonaceous (NC) and carbonaceous (CC) meteorite reservoirs.^36,37^ These include the CC-type iron meteorites Kumerina (group IIC; ∼100 μg per g Ge; Ge/Ni_CI_ ∼0.3) and Needles (group IID; ∼100 μg per g Ge; Ge/Ni_CI_ ∼0.3), as well as the NC-type iron meteorite Henbury (group IIIAB; ∼35 μg per g Ge; Ge/Ni_CI_ ∼0.15). The variable degrees of volatile element depletion (*e.g.*, Ge-depletion) and/or the distinct provenances among these iron meteorites could potentially have affected the Ge stable isotope composition of the samples, making them promising candidates for evaluating natural Ge isotope variations.

In addition to the magmatic iron meteorites, we also analyzed five non-magmatic iron meteorites. Of these, two belong to the IIE irons (NC-type) and three belong to the IAB irons (NC-type). The origin of non-magmatic iron meteorites is often linked to small(er)-scale impact events on planetary surfaces, rather than to the global melting events that characterize the formation of the magmatic irons.^38,39^ Evaporation and (re-)condensation processes during impact-heating related to these local incidents, therefore, might have led to Ge stable isotope variations among these iron meteorite samples.

Finally, some of the samples mentioned above have already been investigated in prior studies.^16,17^ This allows for a direct comparison of the Ge concentrations and stable isotope compositions obtained here to the results of previous studies.

### 3.3 Sample preparation and chemical separation of Ge

#### 3.3.1 Metal samples

For the iron meteorites in this study, ∼100 mg pieces were cut from larger hand specimens using a diamond saw. Special care was taken to sample only pieces with a ‘metallic’ appearance and to avoid darkish, altered, potentially sulfidic areas. Any existing fusion crust was removed and the cutting areas were polished with SiC before the samples were cleaned in an ultrasonic bath in ethanol for 15 min. The cleaned metal pieces were weighed into 15 ml Savillex PFA vials, mixed with appropriate amounts of ^70^Ge–^73^Ge double spike to closely approach the optimal double spike–sample ratio of ∼1 : 1, and digested on a hotplate using 10 ml concentrated HNO_3_ at 120 °C for five days. For all samples, the actual spike–sample mixtures range from 45–55% double spike, which is close to the optimum double spike–sample ratio (see Fig. 1 and 2). As for the iron meteorites, the two metal reference materials NIST 129c and NIST 361, ∼30 mg of metal splinters were weighed into 15 ml Savillex PFA vials, spiked, and digested on a hotplate using 10 ml concentrated HNO_3_ at 120 °C for three days. After complete digestion, the sample solutions were dried down twice at 120 °C and re-dissolved in 0.5 M HNO_3_ for ion exchange chemistry.

The chemical separation of Ge from metal samples *via* ion exchange chemistry followed previously established analytical protocols.^16,17,22,23^ The metal samples were loaded in 0.5 M HNO_3_ onto Bio-Rad columns filled with 2 ml of pre-cleaned and conditioned Bio-Rad AG 50W-X8 cation exchange resin (200–400 mesh, hydrogen form). While Ge was directly eluted with an additional 8 ml 0.5 M HNO_3_, the matrix elements remained on the resin. Small aliquots (1%) were removed from the Ge cuts and cross-checked for purity using a Thermo Scientific iCAP TQ quadrupole inductively coupled plasma mass spectrometer (ICP-MS) at the Max Planck Institute for Solar System Research (MPS) in Göttingen. The samples were introduced into the mass spectrometer in a 0.5 M HNO_3_ solution, and the Ge concentrations were measured in SQ mode, relative to an in-house standard solution. If sufficiently clean for Ge isotope measurements (*i.e.*, Zn/Ge < 0.01, see section 4.3), the Ge cuts were dried down at 120 °C, treated with cHNO_3_ to destroy organic compounds from the cation resin, and re-dissolved in 2 ml 0.5 M HNO_3_. Otherwise, the ion exchange chemistry was repeated once.

#### 3.3.2 Silicate samples

For the terrestrial basalts BHVO-2 and BCR-2, ∼100 mg aliquots were taken from homogeneous bulk powders, weighed into 60 ml Savillex PFA vials, and mixed with appropriate amounts of ^70^Ge–^73^Ge double spike. The samples were digested *via* table-top digestion on a hotplate using 20 ml HF–HNO_3_ (2 : 1) at 120–130 °C (5 days), followed by multiple dry downs in concentrated HNO_3_ at 120 °C to destroy any fluorides that may have formed during the first digestion step. The samples were then dried down twice in 1 M HF at 120 °C and finally re-dissolved in 1–2 ml 1 M HF for ion exchange chemistry.

**Fig. 3:**
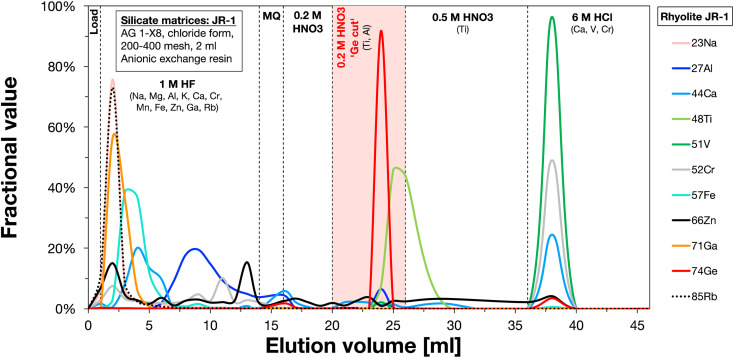
Elution profile of the first step of the ion exchange chromatography (AG 1-X8 anion exchange resin, 200–400 mesh, chloride form) used for the separation of Ge from silicate samples. The rhyolite geostandard JR-1 was doped with 2000 ng Ge prior to loading. Titanium, Al, and other matrix elements partly eluted together with Ge are separated in a second step using the cation exchange chromatography as performed for metal matrices. The elution profile for this chemistry is not shown, as it merely includes the elution of Ge directly after loading, whereas the other elements (*e.g.*, Ti and Fe) remain on the column.

The chemical separation of Ge from the silicate matrices *via* a two-stage ion exchange chemistry was adapted from previously established protocols^16,17,22,23^ and calibrated using the rhyolite JR-1 obtained from the Geological Survey of Japan (Fig. 3). In the first stage, the samples were loaded in 1 ml 1 M HF onto Bio-Rad columns filled with 2 ml of pre-cleaned and conditioned Bio-Rad AG 1-X8 anion exchange resin (200–400 mesh, chloride form), and most matrix elements were directly washed off with an additional 13 ml 1 M HF, whereas Ge together with Ti and some Ca, Al, Mg, and V remained on the column (Fig. 3). After rinsing with 2 ml ultrapure water and 4 ml 0.2 M HNO_3_, Ge was eluted with 6 ml 0.2 M HNO_3_ together with some remaining Al and Ti. The Ge cuts were dried down at 120 °C and re-dissolved in 2 ml 0.5 M HNO_3_. The purification of Ge from Al, Ti, and other remaining matrix elements (especially Zn) was achieved with a second ion chromatography step, which uses Bio-Rad columns filled with 2 ml of pre-cleaned and conditioned Bio-Rad AG 50W-X8 cation exchange resin (200–400 mesh, hydrogen form). The samples were loaded in 2 ml 0.5 M HNO_3_ and Ge was directly eluted with an additional 8 ml 0.5 M HNO_3_, whereas Al, Ti, Zn, and other remaining matrix elements remained on the column. The final Ge cuts were dried down at 120 °C, treated with cHNO_3_ to destroy organic compounds from the cation resin, and then re-dissolved in 2 ml 0.5 M HNO_3_ for Ge isotope measurements.

The yields for each of the two ion exchange procedures were typically >90%. As the Ge double spike is added prior to sample digestion, these slightly incomplete yields are inconsequential for the Ge isotope measurements. Total procedural blanks as measured by isotope dilution are typically less than 1 ng and negligible for all samples, given that >1000 ng Ge was processed for each sample.

### 3.4 Germanium isotope measurements and data reduction

#### 3.4.1 Mass spectrometry

The Ge stable isotope measurements were performed on two different instruments, namely a ThermoScientific Neptune Plus MC-ICP-MS at the Institut für Planetologie, University of Münster and a ThermoScientific Neoma MC-ICP-MS at the Max Planck Institute for Solar System Research in Göttingen. Table 1 summarizes the operating and measurement conditions of the mass spectrometers and Table 2 gives an overview of the Faraday cup settings used on the two instruments.

**Table 1:** Instrument operating and measurement conditions

Instrument operating conditions	Desolvator (Neoma)	Hydride generator (Neoma)	Desolvator (Neptune)	Hydride generator (Neptune)
RF plasma power	1400 W	1400 W	1200 W	1200 W
Plasma gas flow rate	15 l min^−1^	15 l min^−1^	16 l min^−1^	16 l min^−1^
Sampler cone	Standard nickel	Standard nickel	Standard nickel	Standard nickel
Skimmer cone	X nickel	X nickel	X nickel	X nickel
Acceleration voltage	∼10 000 V	∼10 000 V	∼10 000 V	∼10 000 V
Instrumental resolution	Low resolution	Low resolution	Low resolution	Low resolution
Mass analyzer pressure	∼2 × 10^−9^ mbar	∼2 × 10^−9^ mbar	∼4 × 10^−8^ mbar	∼4 × 10^−8^ mbar
Detector	11 Faraday detectors^a^	11 Faraday detectors^a^	9 Faraday detectors^b^	9 Faraday detectors^b^
Sample uptake rate	∼70 μl min^−1^	∼210 μl min^−1^	∼70 μl min^−1^	∼320 μl min^−1^

**Measurement parameters**				
Sample gas flow rate	∼0.75–0.80 l min^−1^	∼0.645 l min^−1^^c^	∼0.75 l min^−1^	∼0.130 l min^−1^^c^
Auxiliary gas flow rate	∼0.75–0.85 l min^−1^	∼0.80 l min^−1^	∼0.70 l min^−1^	∼1.50 l min^−1^
Solution concentration	100 ppb	20 ppb	100 ppb	20 ppb
Typical sensitivity	100 V ppm^−1^	300 V ppm^−1^	60 V ppm^−1^	250 V ppm^−1^
Sample measurement time	50 × 8 s integrations	50 × 8 s integrations	40 × 8.4 s integrations	40 × 8.4 s integrations
Washout time	3.5 min	15 min	3.5 min	15 min
Background measurement time	20 × 8 s integrations	20 × 8 s integrations	20 × 4.2 s integrations	20 × 4.2 s integrations

a All Faraday detectors are connected to 10^11^ Ω feedback resistors.

b The both outer Faraday detectors (L4 and H4) are connected to 10^12^ Ω feedback resistors, while the seven inner Faraday detectors (L3 to H3 as well as the center cup) are connected to 10^11^ Ω feedback resistors.

c Nebulizer gas flow rate.

**Table 2:** Cup settings for Ge stable isotope measurements^a^

Mass spectrometer	L5	L4	L3	L2	L1	C	H1	H2	H3	H4	H5
Neptune	—	^68^Zn	^69^Ga	^70^Ge	^71^Ga	^72^Ge	^73^Ge	^74^Ge	^76^Ge	^77^Se	—
Neoma	^66^Zn	^67^Zn	^69^Ga	^70^Ge	^71^Ga	^72^Ge	^73^Ge	^74^Ge	^75^As	^76^Ge	^77^Se

a Note that the Neptune in Münster only had 9 Faraday cups instead of the 11 Faraday cups of the Neoma in Göttingen.

For both instruments, sample solutions were introduced into the mass spectrometer using either a Savillex C-Flow PFA nebulizer (∼70 μl min^−1^ uptake rate) connected to a Cetac Technologies Aridus II desolvator (hereafter AR) or a Teledyne Cetac Technologies HGX-200 Hydride Generator (hereafter HG). For the AR measurements on the Neoma, the oxide formation was minimized by the addition of N_2_ to the sample gases, resulting in oxide formation rates below 1–2% (measured as Ce/CeO). On the Neptune, however, N_2_ could not be used to minimize oxide production because large quantities of ^40^ArN_2_ would form, which directly interferes with ^68^Zn. Given the Faraday cup configuration on the Neptune, ^68^Zn is the only monitor available to correct for isobaric ^70^Zn interferences on ^70^Ge (note that this is not a problem for the Neoma, where the cup configuration allows monitoring ^66^Zn and ^67^Zn). To reduce oxide production on the Neptune, other parameters such as the auxiliary gas flow rate and torch position were adjusted. For the HG measurements, a 0.25 M NaOH–0.27 M NaBH_4_ solution was freshly prepared before each measurement sequence and mixed with the sample solutions in the reaction chamber of the HG to produce Ge hydrides, which were then introduced into the mass spectrometer.

On both instruments, a combination of standard sampler and X skimmer cones was used and measurements were performed in low resolution mode. Using the AR setup, a signal intensity of ∼10 V on ^70^Ge (^70^Ge is the most abundant Ge isotope in the optimal ∼50 : 50 double spike–sample mixture) was obtained for a ∼100 ppb Ge solution at a ∼70 μl min^−1^ uptake rate. For the HG setup, ∼6 V on ^70^Ge was obtained for a ∼20 ppb Ge solution at a ∼210 μl min^−1^ uptake rate. Ion beams were collected simultaneously using Faraday cups (9 cups covering the mass range ^68^Zn to ^77^Se on the Neptune, 11 cups covering the mass range ^66^Zn to ^77^Se on the Neoma) connected to 10^11^ Ω feedback resistors for all five Ge isotopes (^70^Ge, ^72^Ge, ^73^Ge, ^74^Ge, and ^76^Ge) and the two interference monitors ^66^Zn (Neoma) or ^68^Zn (Neptune) and ^77^Se. On the Neoma, each analysis consisted of on-peak background measurements with 20 × 8 s integrations on a solution blank, followed by 50 cycles of isotope measurements of 8 s each. On the Neptune, on-peak background measurements consisted of 20 × 4.2 s integrations on a solution blank, followed by 40 cycles of isotope measurements of 8.4 s each.

#### 3.4.2 Data reduction

The measured raw data were reduced off-line following a three-dimensional geometric data reduction scheme,^35^ analogously to the method described for Te isotope measurements.^40^ Before the double spike inversion, isobaric interferences from Zn (on ^70^Ge) and Se (on ^74^Ge and ^76^Ge) were iteratively corrected off-line. To this end, measured ion beam intensities on ^66^Zn (Neoma) or ^68^Zn (Neptune) and ^77^Se were converted to raw ^70^Zn and ^74^Se intensities using the natural isotope ratios ^70^Zn/^68^Zn, ^70^Zn/^66^Zn, and ^74^Se/^77^Se and the instrumental mass fractionation factor *β* as estimated from the double spike inversion of the raw ion beam intensities. The raw ^70^Zn and ^74^Se intensities were then subtracted from the measured raw intensities at masses 70 and 74 to obtain interference-corrected (but not mass fractionation-corrected) ^70^Ge and ^74^Ge intensities. Since the instrumental mass fractionation factor *β* used may not be accurate (because it is based on ion beam intensities not corrected for potential interferences), this procedure is repeated iteratively until the calculated value for *β* does not change anymore, which is the case after no more than five iterations.^35^ Overall, the interference corrections were either insignificant or sufficiently low that accurate corrections are possible (see below). Using the interference-corrected ^72^Ge/^70^Ge, ^73^Ge/^70^Ge, and ^74^Ge/^70^Ge ratios, the double spike inversion then provides the natural fractionation factor *α*, for which the Ge stable isotope composition of a sample can be calculated using the following equation:

1 *δ*^74/70^Ge_sample_ = −1000 × (*α*_sample_ − *α*_Bracketing NISTSRM3210a_) × ln(*m*_74_/*m*_70_),

where *m*_74_ and *m*_70_ are the atomic weights of ^74^Ge and ^70^Ge and *α*_Bracketing NISTSRM3210a_ is the mean natural fractionation factor *α* determined for a spiked, bracketing NIST SRM 3210a standard solution measured at similar concentrations before and after each sample. Here, we define *δ*^74/70^Ge as the permil deviation of the ^74^Ge/^70^Ge ratio of a sample from the composition of the NIST SRM 3210a Ge standard:2
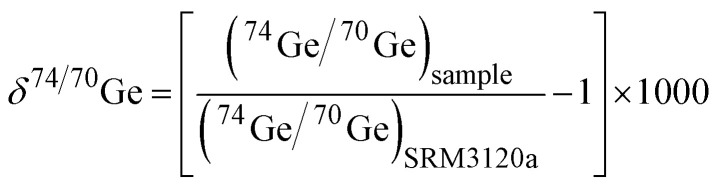


The results for the standards and samples are reported as the mean of replicate measurements and the corresponding errors are Student-*t* 95% confidence intervals (95% CI) for samples with *N* > 3, or the long-term external reproducibility (2 s.d.) of the Ge Alfa Aesar standard solution measurements for samples with *N* ≤ 3.

## 4 Results and discussion

### 4.1 Calibration of the Ge double spike

For the calibration of the ^70^Ge–^73^Ge double spike, the Ge concentrations and isotopic compositions of the ^70^Ge and ^73^Ge single spikes, and the final ^70^Ge–^73^Ge double spike were determined. The Ge concentrations were measured by isotope dilution relative to the certified Ge concentration of the NIST SRM 3120 Ge solution standard. The concentrations determined for the Ge double spike stock solution using nine different standard-double spike mixtures agree with each other with an average concentration of 85.65 ± 0.03 μg g^−1^ (2 s.d.). The Ge isotopic compositions of the single spikes and the double spike were measured relative to the NIST SRM 3120 Ge solution standard, where the ^74^Ge/^70^Ge ratio measured for the standard before and after each spike analysis was used for the correction of instrumental mass fractionation of the measured isotope ratios of the spikes. The isotopic compositions of the single spikes and the double spike are summarized in Table 3.

**Table 3:** Isotopic compositions (in %) of the ^70^Ge and ^73^Ge single spikes and the ^70^Ge–^73^Ge double spike^a^

Mass spectrometer	^70^Ge	^72^Ge	^73^Ge	^74^Ge	^76^Ge
^70^Ge single spike	98.45 (11)	0.562 (9)	0.173 (66)	0.592 (21)	0.131 (11)
^73^Ge single spike	0.68 (17)	1.99 (27)	94.62 (97)	2.36 (56)	0.34 (3)
^70^Ge–^73^Ge double spike	71.5028 (24)	0.9920 (11)	26.2724 (23)	1.0559 (16)	0.1769 (12)

a The ^70^Ge and ^73^Ge single spikes were mixed together in a ratio of 73 : 27, close to the calculated optimum (see Fig. 1). Uncertainties are given in parentheses and refer to last significant digits.

**Fig. 4:**
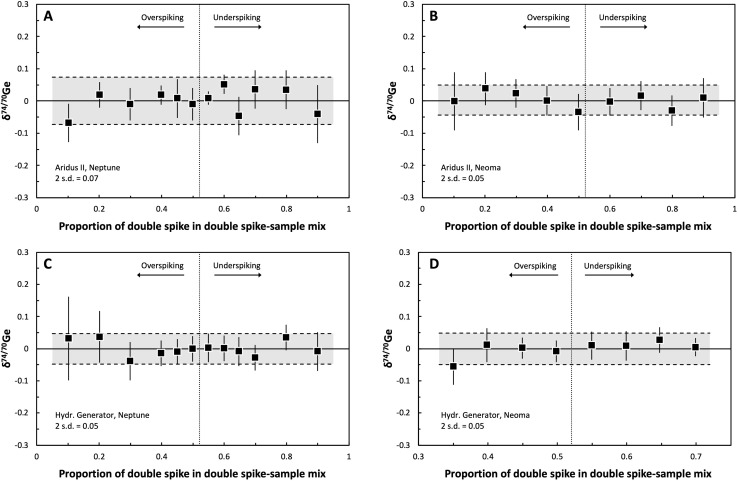
Diagrams of the proportion of double-spike in variable double spike–sample mixtures *vs.* the measured Ge isotopic composition using the Cetac Technologies Aridus II desolvator (AR; panels A and B) or the Teledyne Cetac Technologies HGX-200 Hydride Generator (HG; panels C and D) as sample introduction systems to the Neptune Plus (A and C) or Neoma (B and D) MC-ICP-MS, respectively. In each case the different double spike–sample mixtures define a near-horizontal array, illustrating that (moderate) under- or overspiking has no effect on the analyses and that precise Ge isotope measurements are obtained for the different solution introduction systems and mass spectrometer instruments used. Data points represent the mean of five repeated measurements of each double spike–sample mixture, and error bars correspond to the Student-*t* 95% confidence intervals (CI). The bright grey bands refer to the external reproducibility of each measurement routine (2 s.d.). The dashed vertical lines depict the optimal spike–sample mixing ratio (*f* = 0.5204) as calculated using the Double Spike Toolbox.^29^

The accuracy of the double spike calibration was tested by measuring twelve double spike–standard mixtures with double spike fractions varying between ∼10 and ∼90% (the optimal spike proportion is 52.15%) using both the Neptune and Neoma, and using both the AR and HG setups on both instruments. The double spike–standard mixtures were equilibrated in closed Savillex PFA vials with HNO_3_–HF on a hotplate at 120 °C. For the final double spike calibration, the isotopic compositions of the double spike and the NIST SRM 3120a Ge solution standard were iteratively adapted within their measured uncertainties, to allow for optimal agreement among the Ge isotopic compositions determined for the various double spike–standard mixtures. As shown in Fig. 4, the *δ*^74/70^Ge values obtained for the different spike–standard mixtures are indistinguishable within 0.05–0.07‰ (2 s.d.; depending on the measurement setup) for all four setups (*i.e.*, AR-Neptune, AR-Neoma, HG-Neptune, and HG-Neoma). This together with the constancy of the *δ*^74/70^Ge values over a wide range of spike-to-sample ratios indicates that the double spike calibration is accurate.

### 4.2 Accuracy and reproducibility of Ge double spike measurements

We performed several tests to assess the accuracy of the Ge double spike isotope measurements. First, a split of the Alfa Aesar Ge solution standard has been processed through the entire analytical procedure, including spiking, sample digestion, and chemical separation of Ge. The *δ*^74/70^Ge value measured for this processed standard is indistinguishable from the value measured for the unprocessed standard (Table 3), demonstrating that our analytical protocol does not induce any analytical artifacts on the measured Ge isotope composition.

**Fig. 5:**
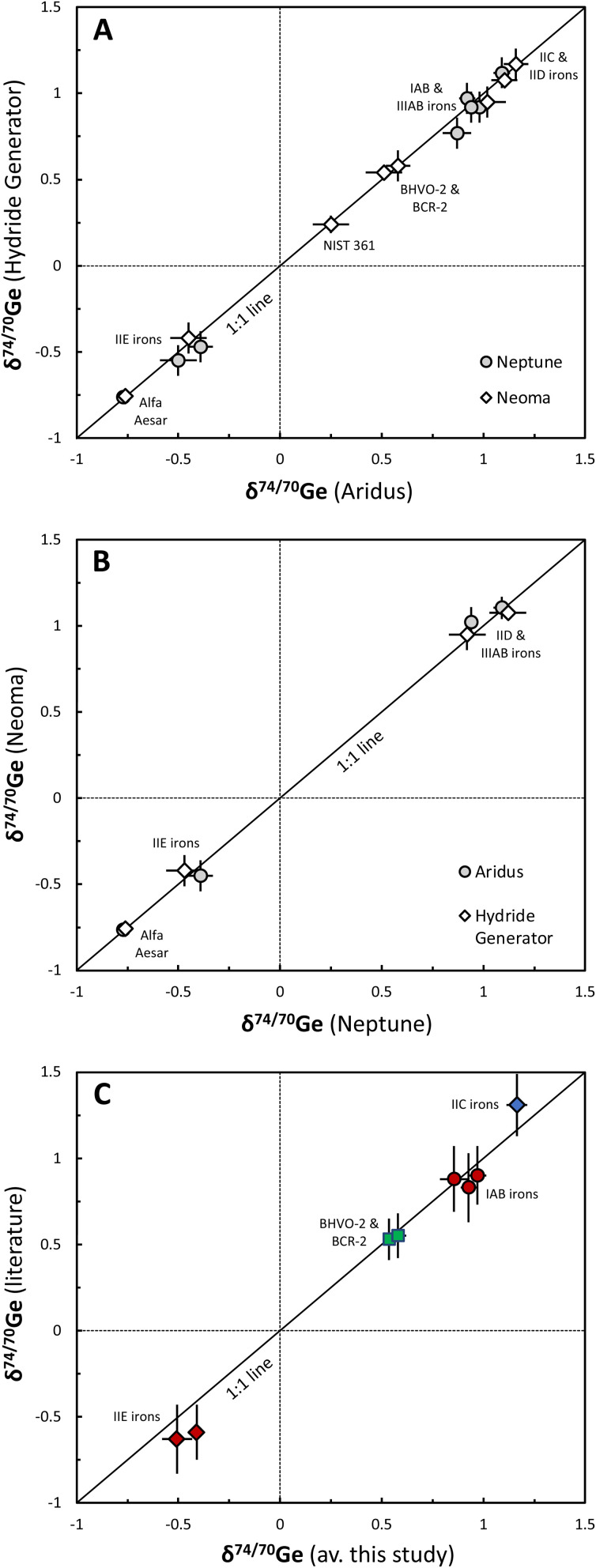
Comparison of the *δ*^74/70^Ge data obtained for different iron meteorite samples, terrestrial basalts, metal reference materials, and the Alfa Aesar Ge solution standard analyzed in this study using (A) either the AR desolvator or the HG setup, or (B) either the Neptune or Neoma MC-ICP-MS. In both cases, all samples align along a 1 : 1 line, indicating that both introduction systems and mass-spectrometers yield consistent results. Error bars represent Student-*t* 95% confidence intervals (CI). (C) Comparison of the average *δ*^74/70^Ge data obtained for different non-carbonaceous (red) and carbonaceous (blue) iron meteorite samples and terrestrial basalts (green) analyzed in this study compared to literature data, using the HG or a spray chamber as the sample introduction system. References are given in Table 4. Overall, the samples align along a 1 : 1 line, indicating that the results of this study are consistent with previous studies. Error bars represent Student-*t* 95% confidence intervals (CI). Note the increase in precision in this study compared to previously reported results.

Second, we analyzed two terrestrial basalts (BHVO-2 and BCR-2) and several iron meteorite samples, which have already been analyzed in prior studies.^9,15,17,18^ These previous studies did not use a double spike, so these data also provide a useful comparison of different analytical methods for Ge isotope measurements. The results of this study are in very good agreement with previously reported results (Table 3, Fig. 5 and 8), demonstrating good overall agreement among the different analytical methods.

The typical external reproducibility (2 s.d.) of the NIST SRM 3120a standard measurements performed during a given measurement session is ±0.08‰ (2 s.d.) for *δ*^74/70^Ge. This uncertainty is determined *via* standard–sample bracketing, where the average composition of the NIST SRM 3120a standard measurements during one session, *α*^Mean^_SRM_, is subtracted from each individual standard measurement in the same session:

3 *δ*^74/70^Ge_sample_ = −1000 × (*α*_SRM_ − *α*^Mean^_SRM_) × ln(*m*_74_/*m*_70_)

**Fig. 6:**
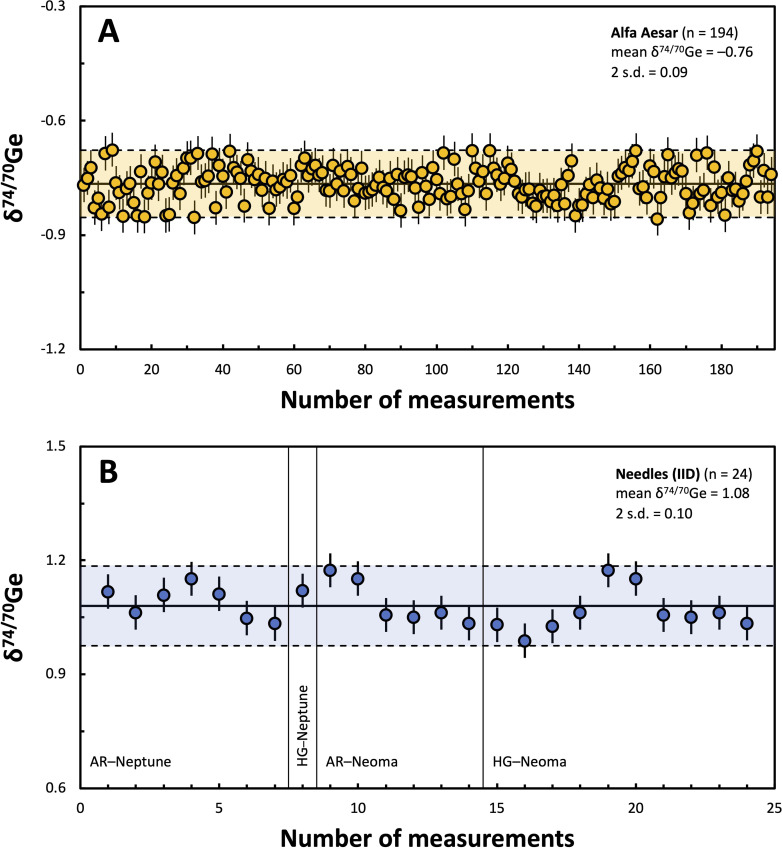
(A) Long-term reproducibility (2 s.d.) of the *δ*^74/70^Ge measurements of the optimally-spiked Ge Alfa Aesar standard solution over a period of two years (*N* = 194). The data points are arranged in the temporal order and include all measurements using the AR-Neptune, HG-Neptune, AR-Neoma, and HG-Neoma setups, respectively. Due to the temporally variable use of either of these setups, the data are not shown individually per setup. However, as seen in Table 4, the mean *δ*^74/70^Ge and corresponding external reproducibility of each measurement setup are indistinguishable from each other. Error bars on the individual data points represent the internal error (2 s.e.) of each measurement (*e.g.*, typically ±0.045). (B) External reproducibility of repeated *δ*^74/70^Ge measurements of the IID iron meteorite Needles (*N* = 24) using the AR-Neptune, HG-Neptune, AR-Neoma, and HG-Neoma setups, respectively, indicating that potential interfering matrix effects as well as sample processing through our analytical protocol do not compromise the accuracy and reproducibility of the Ge isotope measurements.

Repeated analyses of the Ge Alfa Aesar solution standard measured during the course of this study yielded a mean *δ*^74/70^Ge value of −0.76 ± 0.09‰ (*N* = 194; 2 s.d.; Fig. 6), defining a similar external reproducibility as the individual NIST SRM 3120a measurements. To account for potential effects induced by the chemical separation of Ge and by the presence of matrix elements in the analyzed Ge cuts, it is useful to compare the external reproducibility (2 s.d.) obtained for pure standard solutions to that of natural samples. The IID iron meteorite Needles has been measured 24 times during the course of this study using both the AR and HG setups on the Neptune and Neoma instruments. All four different measurement setups yield indistinguishable *δ*^74/70^Ge values and similar external reproducibilities, and the total external reproducibility of the combined measurements is ±0.10‰ for *δ*^74/70^Ge (2 s.d., Table 4), similar to the long-term external reproducibilities obtained for the pure solution standards. This good agreement indicates that sample processing and different sample matrices have no significant effect on the precision of the Ge isotope measurements, and that a value of ±0.09‰ (2 s.d.) provides a good estimate for the external precision of our *δ*^74/70^Ge measurements.

**Table 4:** Germanium concentration and isotopic data for iron meteorites, terrestrial samples, and standard reference materials

Sample	Classification	Ge^a^ (μg g^−1^) (±2σ)	N	*δ* ^74/70^Ge (±2σ)	N	*δ* ^74/70^Ge (±2σ)	N	*δ* ^74/70^Ge (±2σ)	N	*δ* ^74/70^Ge (±2σ)	N	*δ* ^74/70^Ge (±2σ)	*δ* ^74/70^Ge (±2σ)	References
			Aridus II @ Neptune		Hydride Generator @ Neptune		Aridus II @ Neoma		Hydride Generator @ Neoma		Average (this study)		Literature data	
**Iron meteorites**														
C. Diablo	IAB	301.8 ± 3.3	7	0.87 ± 0.07	1	0.77 ± 0.09					8	0.86 ± 0.07	0.88 ± 0.19	1 b
Odessa	IAB	244.3 ± 2.8	6	0.98 ± 0.05	1	0.92 ± 0.09					7	0.97 ± 0.04	0.90 ± 0.17	1 b
Odessa	IAB												0.96 ± 0.07	2
Landes	IAB	366.6 ± 4.0	6	0.92 ± 0.04	1	0.97 ± 0.09					7	0.93 ± 0.04	0.83 ± 0.20	1 b
Kumerina	IIC	97.0 ± 1.1					6	1.16 ± 0.06	1	1.17 ± 0.09	7	1.16 ± 0.05	1.31 ± 0.18	1 b
Needles	IID	96.4 ± 0.9	7	1.09 ± 0.04	1	1.12 ± 0.09	6	1.09 ± 0.06	10	1.06 ± 0.04	24	1.08 ± 0.02		
Miles	IIE	69.1 ± 0.8	6	−0.39 ± 0.06	1	−0.47 ± 0.09	1	−0.45 ± 0.09	1	−0.42 ± 0.09	9	−0.41 ± 0.04	−0.59 ± 0.16	1 b
W. Station	IIE	68.1 ± 0.8	6	−0.50 ± 0.09	1	−0.55 ± 0.09					7	−0.51 ± 0.07	−0.63 ± 0.20	1 b
Henbury	IIIAB	35.4 ± 0.4	5	0.94 ± 0.01	2	0.92 ± 0.09	1	1.02 ± 0.09	1	0.95 ± 0.09	9	0.95 ± 0.02		

**Terrestrial standards**														
BHVO-2	Basalt	1.63 ± 0.02					1	0.51 ± 0.09	5	0.54 ± 0.03	6	0.53 ± 0.03	0.53 ± 0.12	2 and 3
BCR-2	Basalt	1.57 ± 0.02					3	0.58 ± 0.09	1	0.58 ± 0.09	4	0.58 ± 0.04	0.55 ± 0.13	2, 4 and 5

**Solution standard**														
Alfa Aesar			67	−0.77 ± 0.09	9	−0.76 ± 0.08	104	−0.76 ± 0.09	14	−0.76 ± 0.08	194	−0.76 ± 0.09		
Alfa Aesar (proc.)^c^							5	−0.75 ± 0.02			5	−0.75 ± 0.02		

**Metal reference materials**														
NIST 129c	High-S steel	26.3 ± 0.3					7	−0.90 ± 0.04			7	−0.90 ± 0.04		
NIST 361	Steel	32.0 ± 0.4					2	0.25 ± 0.09	5	0.24 ± 0.05	7	0.24 ± 0.03		

a The *δ*^74/70^Ge data of individual samples are reported as the mean of pooled measurements and the corresponding uncertainties reflect Student-*t* 95% confidence intervals (95% CI) for samples with *N* > 3 or the long-term external reproducibility (2 s.d.) of the Ge Alfa Aesar standard solution measurements of 0.09‰ for samples with *N* ≤ 3. Stated uncertainties on the *δ*^74/70^Ge data for the Ge Alfa Aesar standard solution measurements represent two times the standard deviation of the respective measurements. *N*: Number of Ge isotope analyses. Germanium concentrations as determined by isotope dilution (*e.g.*, Stracke and Scherer, 2014;^30^ see the main text for details). References: (1) Luais *et al.*, 2007;^16^ (2) Escoube *et al.*, 2012;^18^ (3) Rouxel and Luais, 2017;^9^ (4) Rouxel *et al.*, 2006;^15^ (5) Luais *et al.*, 2012.^17^

b Re-normalized to the Ge SRM 3120a solution standard by Luais *et al.* (2012).

c This Alfa Aesar solution has been processed together with the samples through the entire analytical protocol, including sample digestion, chemical separation of Ge, and isotope measurements.

### 4.3 Influence of isobaric interferences on Ge double spike measurements

Isobaric interferences might compromise the accuracy and precision of the Ge isotope measurements. Monoatomic isobaric interferences on the Ge isotopes used in the double spike inversion scheme may arise from the presence of ^70^Zn (on ^70^Ge) and ^74^Se (on ^74^Ge). Fortunately, however, ^70^Ge and ^74^Ge are the two most abundant Ge isotopes in optimal spike–sample mixtures (*i.e.*, ∼47% and ∼18%, respectively), while ^70^Zn and ^74^Se are the least abundant isotopes of their elements (*i.e.*, 0.6% and 0.9%, respectively). Selenium is very efficiently removed during the chemical purification of Ge (all final Ge cuts of this study had Se/Ge < 1 × 10^−4^ and resulting ^74^Se/^74^Ge < 1 × 10^−5^) and is also suppressed during isotope measurements using the AR setup by use of HNO_3_ as the running solution. As such, isobaric interferences from Se are insignificant for the Ge isotope measurements.

Similarly, for the HG setup, Zn is efficiently removed because it does not form hydrides and, consequently, does not affect the Ge isotope measurements. However, for the AR setup isobaric Zn interferences may be relevant. We, therefore, tested the tolerable amount of Zn in the measured Ge solutions for reliable correction and accurate measurements using the AR setup by a series of Zn doping tests, in which variable amounts of Zn were added to the spiked NIST SRM 3210a Ge standard solution. The final Ge cuts after the chemical purification of Ge had Zn/Ge < 0.005, hence, we performed a Zn doping test in the Zn/Ge range between 0.00025 and 0.11. The results of the doping tests show that the presence of Zn can be accurately corrected for up to Zn/Ge ratios of 0.01, which is much higher than the range of Zn/Ge observed for the final Ge cuts of the samples.

**Fig. 7:**
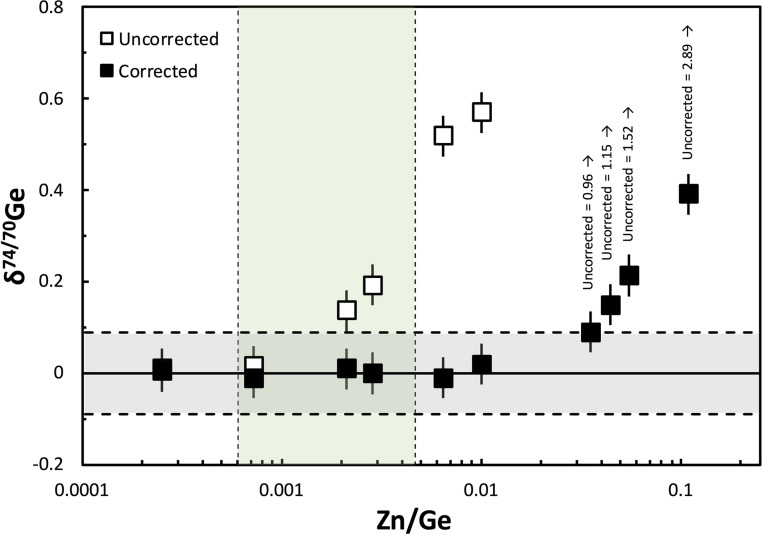
Plot of *δ*^74/70^Ge *vs.* Zn/Ge, illustrating the effect of isobaric Zn interferences (on ^70^Ge) on the measured *δ*^74/70^Ge composition of variably Zn-doped, optimally spiked NIST SRM 3120a Ge solution standard mixtures using the AR as the sample introduction system to the Neoma MC-ICP-MS. The horizontal grey band represents the long-term external reproducibility of our measurement routine of 0.09‰ (Fig. 6), and the vertical green band represents the total range of Zn/Ge of the processed standard reference materials, natural samples, and standard solutions analyzed in this study. In the range of relevant Zn/Ge, isobaric Zn interferences are either insignificant or can be corrected accurately. Of note, doping tests using the AR as the sample introduction system to the Neptune MC-ICP-MS yield very similar results. By contrast, Zn doping tests cannot be made using a HG setup, as Zn does not form hydrides and, thus, is efficiently removed using the HG as the sample introduction system. Therefore, the general very good accordance of AR and HG measurements gives further proof for the accurate correction of isobaric Zn interferences using the AR setup.

Further isobaric interferences may arise from oxides and other polyatomic compounds. However, oxide production is irrelevant for HG measurements as all forming oxides of elements that have been hydrogenated before introduction into the mass spectrometer would have higher masses than the Ge isotopes used in the double spike inversion.^41–43^ For the AR setup, oxides might affect the quality of the Ge isotope measurements, but care was taken to minimize oxide production rates. The excellent agreement of measured *δ*^74/70^Ge values using either the AR or HG setup illustrates that the potential production of oxides has no significant effect on the Ge isotope measurements using the AR setup (Fig. 7).

### 4.4 Comparison of Aridus and hydride generator measurements

This study is the first to use an Aridus desolvator as the sample introduction system (*i.e.*, the AR setup) for Ge isotope measurements. Therefore, it is useful to compare the results obtained using the AR setup to those obtained using the hydride generator (*i.e.*, the HG setup), which is the sample introduction system used in some prior studies.^18,22,23^ In general, we find excellent agreement for all samples that have been analyzed with both the AR and HG setups, regardless of whether the measurements were performed on the Neptune or the Neoma MC-ICPMS (Table 3 and Fig. 5). Thus, both the AR and HG setups can equally well be used for accurate and precise Ge stable isotope measurements, where the choice between the two setups will depend on the scope of application, sample type, and availability of sample material.

The major advantage of the HG setup over the AR setup is that volatile aqueous species (*e.g.*, GeOH_4_) are converted to gaseous hydride species (*e.g.*, GeH_4gas_). This effect not only removes isobaric interferences from elements that do not form hydrides (*e.g.*, Zn, FeO, and NiO),^22^ but also results in a strong increase in Ge sensitivity.^9^ Moreover, as noted above, the oxide formation has no effect on the Ge isotope measurements, because Ge is the lightest hydride-forming element in classical HG setups. The major disadvantage of the HG setup over the AR setup is that it is more time-consuming and more complex in handling the measurement setup (*e.g.*, rinse times have to be increased to 15 min to ensure an efficient wash-out of any Ge from the HG tubing; the high uptake rate consumes more chemicals needed in the preparation of the measurement solution; reducing agents (NaOH or NaBH_4_) are needed). Thus, while the HG setup may be the best choice for Ge isotope measurements in samples with low Ge abundances or complex sample matrices, for most samples the AR setup may be the easier and more efficient method for Ge isotope measurements.

### 4.5 Germanium stable isotope variations among natural samples

**Fig. 8:**
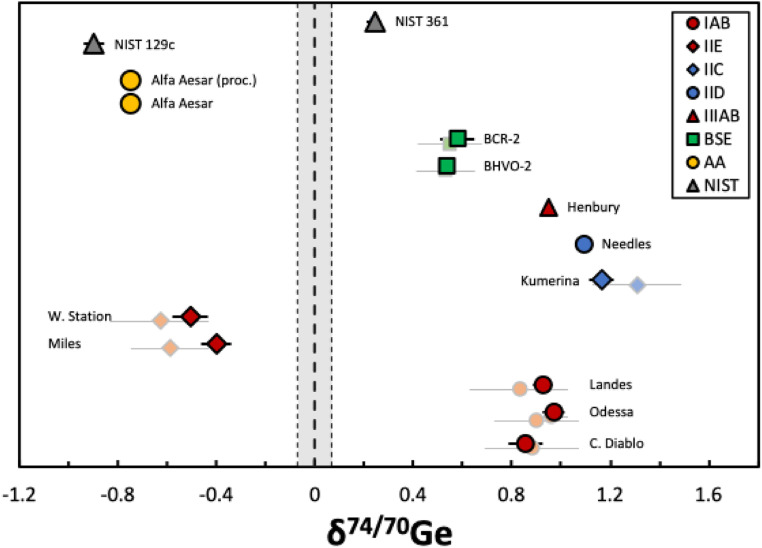
Caltech diagram of the *δ*^74/70^Ge data of the eight iron meteorites (NCs in red and CCs in blue), two terrestrial basalt standards (green), the Ge Alfa Aesar solution standard (yellow), and two metal reference materials (grey) analyzed in this study. Uncertainties of the individual samples are Student-*t* 95% confidence intervals (95% CI). Light colored symbols represent literature data; references are listed in Table 4.

To assess the magnitude and direction of Ge isotope fractionations in natural samples, we analyzed eight iron meteorites, two terrestrial basalts, and two NIST metal reference materials. The Ge concentrations and isotopic compositions measured for all samples (and using different measurement setups) are listed in Table 4 and shown in Fig. 8. The Ge concentrations of the samples were precisely determined by isotope dilution and agree well with literature data.^9,15,17,18,44^ The Ge concentration obtained for NIST SRM 361 is also consistent with data provided in the GeoReM database (to our knowledge there are no literature data available for the NIST SRM 129c standard).

All samples analyzed in this study are isotopically fractionated relative to the NIST SRM 3120a standard and display overall *δ*^74/70^Ge variations of up to ∼2‰. These variations are well-resolved using our Ge double spike method, given that the external uncertainty of our method is 0.09‰ (2 s.d.). The Alfa Aesar Ge solution standard and NIST 129c metal have negative *δ*^74/70^Ge values and, thus, are isotopically lighter than the NIST SRM 3120a standard, while the NIST 361 metal has positive *δ*^74/70^Ge values and, thus, is isotopically heavier than the NIST SRM 3120a standard. These isotopic variations among different reference materials may either be inherited from different starting raw materials (*e.g.*, ores) or are the result of isotope fractionation induced during the standard production. The terrestrial basalts BHVO-2 and BCR-2 show distinctly more positive *δ*^74/70^Ge values compared to all other reference materials mentioned above. These more positive *δ*^74/70^Ge values are indistinguishable from values reported for other terrestrial igneous rocks,^9,10^ suggesting that high-temperature magmatic processes induce no significant Ge isotope fractionation.

The three magmatic iron meteorites investigated here (*i.e.*, groups IIC, IID, and IIIAB) display even heavier Ge isotope compositions than the terrestrial basalts, in line with observations from previous studies.^16,17^ Moreover, despite having variable Ge concentrations and Ge/Ni ratios, these three samples have very similar *δ*^74/70^Ge values, suggesting that the variable volatile element depletions of their parent bodies did not induce large Ge isotope fractionations. By contrast, the non-magmatic iron meteorites investigated here (*i.e.*, groups IAB and IIE) display variable Ge isotope compositions, including both light and heavy compositions. These samples might have formed by local impacts on planetary surfaces and, thus, might have lost significant amounts of volatile Ge in the course of impact heating.^38,39^ As such, these samples provide evidence for Ge isotope fractionation during localized heating and degassing. Overall, the data of this study demonstrate that large Ge isotope fractionation occur among terrestrial and extraterrestrial samples, making Ge isotopes a powerful tool for a wide range of geochemical and cosmochemical applications.

## 5 Conclusions

We developed and compared different analytical procedures for the combined and precise Ge concentration and mass-dependent isotope measurement of silicate and metal samples using ^70^Ge–^73^Ge double spike MC-ICPMS. Data were obtained using both a Neptune Plus and a Neoma MC-ICP-MS, and using either a Cetac Aridus II desolvator (AR) or a Teledyne Cetac Technologies HGX-200 Hydride Generator (HG) as the sample introduction system, respectively. The double spike was calibrated for all four possible combinations (*i.e.*, AR-Neptune, HG-Neptune, AR-Neoma, and HG-Neoma) and provides accurate results over a wide range of spike-to-sample ratios. This study is the first to use a desolvator for sample introduction (*i.e.* the AR setup), and we find excellent agreement between data obtained using this setup and the more commonly used HG setup. Due to the easier and more efficient use, we recommend the AR setup as the method of choice for most Ge isotope measurements related in geo- and cosmochemistry. The chemical separation procedure of Ge, which includes two-stage ion exchange chromatography for silicate samples and single-stage cation exchange chromatography for metal samples, results in sufficiently clean Ge cuts such that isobaric interferences from any remaining Zn (and Se) are small and can be reliably corrected. The external reproducibility (2 s.d.) of the entire analytical procedure has been determined by repeated measurements of the Ge Alfa Aesar standard solution and the IID iron meteorite Needles and is 0.09‰ for *δ*^74/70^Ge. The iron meteorites, terrestrial basalts, metal reference materials, and the Ge Alfa Aesar standard solution investigated in this study display well-resolved *δ*^74/70^Ge variations of up to ∼2‰, which agree well with literature data, if present. Altogether, the data of this study further demonstrate that Ge stable isotopes are a powerful tracer for disentangling the nature and conditions of a wide range of geochemical and cosmochemical processes.

## Data availability

Data supporting this article/generated as part of this work have been included as part of the tables (and figures) attached to the manuscript.

## Author contributions

Elias Wölfer: investigation, formal analysis, methodology, data curation, validation, visualization, writing – original draft, writing – review & editing. Christoph Burkhardt: conceptualization, funding acquisition, project administration, methodology, resources, supervision, writing – review & editing. Thorsten Kleine: conceptualization, funding acquisition project administration, resources, supervision, writing – review & editing.

## Conflicts of interest

There are no conflicts to declare.
